# Tidal volume challenge to predict preload responsiveness in patients with acute respiratory distress syndrome under prone position

**DOI:** 10.1186/s13054-022-04087-w

**Published:** 2022-07-18

**Authors:** Rui Shi, Soufia Ayed, Francesca Moretto, Danila Azzolina, Nello De Vita, Francesco Gavelli, Simone Carelli, Arthur Pavot, Christopher Lai, Xavier Monnet, Jean-Louis Teboul

**Affiliations:** 1 Service de Médecine Intensive-Réanimation, Hôpital de Bicêtre, Hôpitaux Universitaires Paris-Saclay, INSERM UMR_S999, Université Paris-Saclay, AP-HP, 78 rue du Général Leclerc, 94270 Le Kremlin-Bicêtre, France; 2 Department of Environmental and Preventive Science, University of Ferrara, Ferrara, Italy

**Keywords:** Pulse pressure variation, Fluid responsiveness, ARDS, End-expiratory occlusion test

## Abstract

**Background:**

Prone position is frequently used in patients with acute respiratory distress syndrome (ARDS), especially during the Coronavirus disease 2019 pandemic. Our study investigated the ability of pulse pressure variation (PPV) and its changes during a tidal volume challenge (TVC) to assess preload responsiveness in ARDS patients under prone position.

**Methods:**

This was a prospective study conducted in a 25-bed intensive care unit at a university hospital. We included patients with ARDS under prone position, ventilated with 6 mL/kg tidal volume and monitored by a transpulmonary thermodilution device. We measured PPV and its changes during a TVC (ΔPPV TVC_6–8_) after increasing the tidal volume from 6 to 8 mL/kg for one minute. Changes in cardiac index (CI) during a Trendelenburg maneuver (ΔCI_TREND_) and during end-expiratory occlusion (EEO) at 8 mL/kg tidal volume (ΔCI EEO_8_) were recorded. Preload responsiveness was defined by both ΔCI_TREND_ ≥ 8% and ΔCI EEO_8_ ≥ 5%. Preload unresponsiveness was defined by both ΔCI_TREND_ < 8% and ΔCI EEO_8_ < 5%.

**Results:**

Eighty-four sets of measurements were analyzed in 58 patients. Before prone positioning, the ratio of partial pressure of arterial oxygen to fraction of inspired oxygen was 104 ± 27 mmHg. At the inclusion time, patients were under prone position for 11 (2–14) hours. Norepinephrine was administered in 83% of cases with a dose of 0.25 (0.15–0.42) µg/kg/min. The positive end-expiratory pressure was 14 (11–16) cmH_2_O. The driving pressure was 12 (10–17) cmH_2_O, and the respiratory system compliance was 32 (22–40) mL/cmH_2_O. Preload responsiveness was detected in 42 cases. An absolute change in PPV ≥ 3.5% during a TVC assessed preload responsiveness with an area under the receiver operating characteristics (AUROC) curve of 0.94 ± 0.03 (sensitivity: 98%, specificity: 86%) better than that of baseline PPV (0.85 ± 0.05; *p* = 0.047). In the 56 cases where baseline PPV was inconclusive (≥ 4% and < 11%), ΔPPV TVC_6–8_ ≥ 3.5% still enabled to reliably assess preload responsiveness (AUROC: 0.91 ± 0.05, sensitivity: 97%, specificity: 81%; *p* < 0.01 *vs.* baseline PPV).

**Conclusion:**

In patients with ARDS under low tidal volume ventilation during prone position, the changes in PPV during a TVC can reliably assess preload responsiveness without the need for cardiac output measurements.

*Trial registration: *ClinicalTrials.gov (NCT04457739). Registered 30 June 2020 —Retrospectively registered, https://clinicaltrials.gov/ct2/show/record/NCT04457739

**Supplementary Information:**

The online version contains supplementary material available at 10.1186/s13054-022-04087-w.

## Background

Prone positioning is recommended in mechanically ventilated patients with acute respiratory distress syndrome (ARDS) and a ratio of partial pressure of arterial oxygen (PaO_2_) to the fraction of inspired oxygen (FiO_2_) < 150 mmHg [1–3], for at least 16 h [1] and even more for some patients [4]. During the Coronavirus disease 2019 (COVID-19) pandemic, prone positioning has been widely applied in patients with ARDS [5–9]. Patients with ARDS, especially those with COVID-19, are characterized by lung edema mainly related to increased pulmonary endothelial permeability [10, 11]. Fluid management in such patients is challenging [12, 13]. On the one hand, ARDS patients could experience shock that might require fluid therapy and vasopressors [14, 15]. The objective of fluid therapy is to restore adequate organ perfusion in patients in the case of fluid responsiveness, a phenomenon that is generally present in 50% of patients [16]. On the other hand, fluid therapy may worsen lung edema due to the altered permeability of pulmonary microvessels [17]. Therefore, the prediction of fluid responsiveness is important to test in patients with ARDS to prevent fluid administration in those who are fluid unresponsive and for whom harmful consequences of fluid therapy would be maximal [18]. Pulse pressure variation (PPV), passive leg raising (PLR), and end-expiratory occlusion (EEO) are dynamic variables or tests that are commonly used to predict fluid responsiveness in mechanically ventilated patients [19, 20]. Nevertheless, PLR and EEO test require real-time cardiac output measurements, while PPV, which can be measured non-invasively [21, 22] or invasively by a simple arterial catheter, is less reliable in patients mechanically ventilated with a low tidal volume [23–25]. The tidal volume challenge (TVC) was suggested to compensate for the limitation of PPV during low tidal volume ventilation [26], though limited and controversial evidence exists in patients under prone position [27, 28]. The primary objective of our study was to investigate whether a one-minute TVC could assess preload responsiveness in patients with ARDS under prone position. The secondary objective was to investigate the predictive performance of EEO test at the tidal volume of 6 mL/kg predicted body weight (PBW) under prone position.

## Methods

This is a prospective study conducted in an intensive care unit (ICU) of a tertiary hospital. Our study was approved by Comité de Protection des Personnes (2019-A00064-53) and was registered on ClinicalTrials (NCT04457739).

### Patients

We included patients with ARDS according to the Berlin definition [29], under prone position and monitored by calibrated transpulmonary thermodilution device (PiCCO2, Getinge, Sweden). Patients for whom an assessment of preload responsiveness was required by the attending physician were included. Exclusion criteria were: age ≤ 18 years, pregnancy, presence of cardiac arrhythmia or venous compression stockings, presence of extracorporeal membrane oxygenation (ECMO) assistance at the time of inclusion, and contraindication to the Trendelenburg maneuver. Informed consent was obtained from the patient’s next of kin.

### Transpulmonary thermodilution measurements

In all patients, a thermistor-tipped femoral artery catheter and a central venous catheter were already in place as part of the patient’s hemodynamic monitoring [30]. After calibrating the monitoring system by transpulmonary thermodilution (TPTD) [30], the continuous pulse contour analysis-derived cardiac index (CI) could be estimated [30]. The following TPTD variables were also collected at baseline: cardiac index, global end-diastolic volume indexed for body surface (GEDVI), extravascular lung water index for body weight (EVLWi), and pulmonary vascular permeability index (PVPI).

### Ventilation settings

All the patients received protective ventilation with the settled tidal volume of 6 mL/kg PBW in the volume assist-controlled mode. The respiratory rate and the FiO_2_ were adjusted by the attending physician. Neuromuscular-blocking agents were used if required. Before prone positioning, we collected the blood lactate concentration and the arterial blood gases data, including PaO_2_/FiO_2_. The respiratory system compliance (Crs) was calculated under prone position by calculating the ratio of tidal volume over the difference between plateau pressure and total positive end-expiratory pressure (PEEP) at baseline.

### Other variables

Demographic and other hemodynamic parameters, including heart rate, arterial blood pressure, and central venous pressure (CVP), were recorded. The intra-abdominal pressure (IAP) was recorded at each timepoint under prone position. The fluid balance during the last 24 h before prone positioning was collected. The use of norepinephrine and its dose were recorded.

### Design of the study

**Fig. 1:**
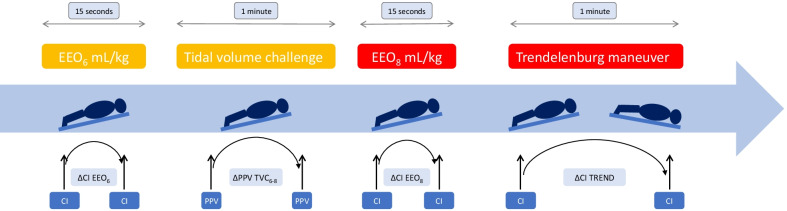
Study protocol

At the time of inclusion, patients were in the prone position with a 13° upward bed angulation [28] (Fig. 1). A set of TPTD measurements was performed, and related hemodynamic and respiratory variables were collected. Then, while tidal volume was at 6 mL/kg PBW, we performed an EEO test (EEO_6_) for 15 s as previously described [31] and the pulse contour analysis CI was recorded at the end of EEO_6_. The percent changes in CI during EEO (ΔCI EEO_6_) were then calculated. After the pulse contour analysis CI returned to the baseline value, we recorded the PPV value (PPV_base_) before increasing the tidal volume from 6 to 8 mL/kg for one minute. We recorded the maximal value of PPV (PPV_max_) during the maneuver and calculated the absolute changes in PPV (ΔPPV TVC_6–8_) during TVC (PPV_max_–PPV_base_). Then, we performed another EEO test for 15 s [16], while the tidal volume was kept at 8 mL/kg (EEO_8_). We calculated the percent changes in pulse contour analysis CI during the second EEO test (ΔCI EEO_8_), and then, we decreased the tidal volume to 6 mL/kg PBW. When the pulse contour analysis CI was stabilized again (CI_base_), we performed a Trendelenburg maneuver by using the automatic moving function of the bed. This resulted in a patient’s position where the head and the trunk were lowered to a maximum of − 13° bed angulation. After one minute, the maximal value of pulse contour analysis CI (CI_max_) was recorded to calculate the percent changes in CI during Trendelenburg (ΔCI_TREND_) = (CI_max _– CI_base_)/CI_base_. According to the decision of the attending physician, some patients received 500 mL of saline over 10 min. In this subgroup of patients, a new set of hemodynamic variables including TPTD measurements was obtained at the end of the saline infusion. Doses of norepinephrine and of sedative drugs, as well as respiratory rate and level of PEEP, were kept constant throughout the study period. Some patients were studied more than once. Nevertheless, in this case, the studies were never performed more than once during the same day or the same session of prone position.

### Statistical analysis

The Trendelenburg test was defined as positive if ΔCI_TREND_ was ≥ 8% according to Yonis et al. [28]. The EEO test was defined as positive if ΔCI EEO_8_ ≥ 5% [32]. Since we did not assess the response to fluid administration in all patients, we defined preload responsiveness as when both ΔCI_TREND_ and ΔCI EEO_8_ were ≥ 8% and ≥ 5%, respectively. Preload unresponsiveness was defined as when both ΔCI_TREND_ and ΔCI EEO_8_ < 8% and < 5%, respectively. Results in terms of preload responsiveness and preload unresponsiveness were given according to cases and not to patients as some patients were studied more than once. Cases, where only ΔCI_TREND_ was ≥ 8% or only ΔCI EEO_8_ was ≥ 5%, were excluded from the final analysis. The main objective of this study was to investigate whether the changes in PPV (ΔPPV TVC_6–8_) during TVC could assess preload responsiveness under prone position with an area under the receiver operating characteristics (ROC) curve (AUROC) of at least 0.9. We considered that the null hypothesis was at 0.75 since an AUROC between 0.5 and 0.75 would have been too low in terms of sensitivity and specificity to draw a relevant conclusion. Considering an *α* risk at 5% and a *β* risk at 10%, and assuming that the prevalence of preload responsiveness is 50% [16]. We thus calculated that 84 cases were required by using the method of Hanley-McNeil [33]. We also tested the performance of PPV_base_, of PPV_max_, and of EEO_6_ to predict preload responsiveness.

Quantitative and categorical variables are presented as frequency (percentage) and mean ± SD or median (interquartile range) as appropriate. Histograms were used to verify the distribution of data. The ROC curve data are presented as the AUROC value ± standard error (with a 95% confidence interval), sensitivity (with a 95% confidence interval), and specificity (with a 95% confidence interval). The *p* values for the interaction effects among different timepoints, between baseline and maximal values, and between responsive cases and non-responsive cases, have been calculated by estimating a Generalized Linear Mixed Effect Model (GLMM). The model considers a random effect term on the repeated measurements accounting for the correlation between repeated measurements. The details of this model are presented in the additional file [34]. Moreover, the Benjamini and Hochberg corrections were performed for the multiple testing correction in Table 3 [35]. The p value adjustment method has been used instead of the false discovery rate definition [36].

The fivefold cross-validated AUROC estimates for a repeated measurements data set have been calculated by using the LeDell approach [37, 38]. A computationally efficient influence curve-based approach was used to obtain a variance estimate for cross-validated AUROC [37]. The stratified bootstrapped *p* value (10,000 runs) was calculated for the comparison with the gold standard of 0.75. Because of repeated measurements, 95% confidence intervals around the parameters were estimated using a 10,000 individual bootstrap by resampling subjects instead of measurements [39]. The same set bootstrapping of samples was used for comparison between AUROCs in each iteration, preserving the correlation between repeated measurements [40].

A *p* value < 0.05 was considered statistically significant. The gray zone analysis has been conducted as reported elsewhere [41, 42]. The Youden index (sensitivity + specificity − 1) was used to define the optimal cutoff values for each test and its calculation was conducted within 10,000 bootstraps resamples. By using a two-step procedure, the gray zone was then defined as the values presenting with either sensitivity less than 90% or specificity less than 90% [42].

Statistical analysis was performed using MedCalc 11.6.0 software (Mariakerke, Belgium), and cutpoint package in R software (version 3.4.1).

## Results

### Patient characteristics

Patients with ARDS ventilated with a 6 mL/kg tidal volume under prone position were prospectively included from January 2019 to May 2021. In total, we included 69 patients of whom 55 had COVID-19 (76%). The inclusions were conducted after prone positioning for 11 (2–14) hours. Data from eleven patients (13 cases) were excluded from the analysis since only one of the two preload responsiveness tests (Trendelenburg or EEO_8_) was positive (three cases with only ΔCI_TREND_ ≥ 8% and ten cases with only ΔCI EEO_8_ ≥ 5%). Therefore, data from 58 patients were included in the analysis (Table 1). Sixteen patients were studied more than once (maximal number: four times), and the delay between two inclusions was 5 (3–14) days. A total of 84 different cases were finally analyzed (Table 2). According to the increase in CI both during Trendelenburg and EEO_8_, preload responsiveness was found in 42 cases and preload unresponsiveness was found in 42 other cases.

**Table 1:** General characteristics of patients with acute respiratory distress syndrome under prone position at the time of inclusion

Variables	All patients (*n* = 58)
Age (years)	65 ± 11
Male (*n*, %)	45 (76)
Body mass index (kg/m^2^)	27.4 (24.5–30.7)
Hypertension (*n*, %)	33 (57%)
COVID-19 (*n*, %)	44 (76)
SAPS II at admission	39 (32–52)
SOFA score at admission	4 (4–6)
ICU length of stay (days)	23 (12–35)
ICU mortality (*n*, %)	32 (55)

Values are expressed as mean ± SD or median (interquartile range) or number (percentage). COVID-19, Coronavirus disease 2019; ICU, intensive care unit; SAPS II, Simplified Acute Physiology Score II; and SOFA, Sequential Organ Failure Assessment

**Table 2:** Baseline cardiovascular and respiratory parameters of all the cases at the time of inclusion

Studied variables	All cases (*n* = 84)	Non-responsive cases (*n* = 42)	Responsive cases (*n* = 42)	*p* value
Inclusion time after admission (days)	7 (4–11)	8 (5–15)	6 (3–10)	0.145
Inclusion time after prone positioning (hours)	11 (2–14)	12 (5–15)	8 (1–12)	**0.033**
*Hemodynamic variables*				
Number of patients with norepinephrine (*n*, %)	70 (83)	35 (83)	35 (83)	0.770
Dose of norepinephrine (µg/kg/min)	0.25 (0.15–0.42)	0.23 (0.14–0.35)	0.32 (0.17–0.56)	0.382
Heart rate (beats/min)	83 (69–100)	82 (67–94)	94 (74–107)	**0.014**
Systolic arterial pressure (mmHg)	127 ± 18	127 ± 18	127 ± 18	1.000
Diastolic arterial pressure (mmHg)	59 ± 10	57 ± 8	60 ± 11	0.131
Mean arterial pressure (mmHg)	82 ± 12	82 ± 11	83 ± 13	0.594
Central venous pressure (mmHg)	10 ± 4	11 ± 5	8 ± 4	**0.012**
Intra-abdominal pressure (mmHg)	11 ± 4	11 ± 4	11 ± 4	0.924
CI_TPTD_ (L/min/m^2^)	3.06 ± 0.90	3.38 ± 0.95	2.73 ± 0.74	**0.001**
GEDVI (mL/m^2^)	744 ± 173	780 ± 202	708 ± 130	0.055
EVLW (mL/kg)	17 (15–20)	17 (15–20)	17 (14–21)	0.936
PVPI	3.4 (2.9–4.1)	3.2 (2.6–4.1)	3.5 (2.9–4.1)	0.293
Fluid balance of previous 24 h (mL)	562 (-224–1660)	500 (-183–1720)	693 (-280–1717)	0.831
Lactate (mmol/L)	1.6 (1.3–2.3)	1.5 (1.1–1.8)	2.1 (1.3–2.8)	**0.005**
*Respiratory variables*				
Respiratory rate (breaths/min)	30 (26–33)	30 (28–35)	28 (25–30)	**0.035**
PaO_2_/FiO_2_ before prone positioning (mmHg)	104 ± 27	105 ± 24	102 ± 29	0.684
Tidal volume before TVC (mL/kg PBW)	6.0 (6.0–6.0)	6.0 (6.0–6.0)	6.0 (6.0–6.0)	0.267
Tidal volume during TVC (mL/kg PBW)	8.0 (8.0–8.0)	8.0 (8.0–8.0)	8.0 (8.0–8.0)	0.321
Total PEEP (cmH_2_O)	14 (11–16)	14 (12–16)	14 (10–16)	0.946
Driving pressure before TVC (cmH_2_O)	12 (10–17)	13 (10–18)	12 (10–17)	0.723
Plateau pressure before TVC (cmH_2_O)	27 (25–31)	27 (25–30)	27 (25–31)	0.882
Crs before TVC (mL/cmH_2_O)	32 (22–41)	31 (21–39)	34 (22–41)	0.591
Driving pressure at the end of TVC (cmH_2_O)*	19 (15–22)	19 (15–21)	20 (15–24)	0.473
Plateau pressure at the end of TVC (cmH_2_O)*	32 (29–37)	33 (30–38)	32 (28–35)	0.229
Crs at the end of TVC (mL/cmH_2_O)*	28 (22–39)	28 (24–39)	27 (21–39)	0.715

*CI* cardiac index, *Crs* compliance of the respiratory system, *EVLW* extravascular lung water, *GEDVI* global end-diastolic volume index, *PVPI* pulmonary vascular permeability index, *PaO*_*2*_*/FiO*_*2*_ the ratio of partial pressure of arterial oxygen to fraction of inspired oxygen, *PBW* predicted body weight, *PEEP* positive end-expiratory pressure, *TPTD* transpulmonary thermodilution, *TVC* tidal volume challenge; Bold font indicates statistical significance

*Available in 78 cases

The demographic characteristics of all the 58 analyzed patients are detailed in Table 1. Table 2 shows the detailed general characteristics of all the 84 analyzed cases. The mean arterial pressure was 82 (75–90) mmHg [under norepinephrine in 70 (83%) cases at a dose of 0.25 (0.15–0.42) µg/kg/min] at the time of inclusion. The PEEP was 14 (11–16) cmH_2_O. The driving pressure was 12 (10–17) cmH_2_O before the TVC versus 19 (15–22) cmH_2_O at the end of the TVC (*p* < 0.001), the plateau pressure was 27 (25–31) cmH_2_O before the TVC versus 32 (29–37) cmH_2_O at the end of the TVC (*p* < 0.001), and the Crs was 32 (21–41) mL/cmH_2_O before the TVC versus 28 (22–39) mL/cmH_2_O at the end of the TVC (*p* = 0.366). The evolution of hemodynamic variables in both preload responsive cases and preload unresponsive cases is shown in Table 3. The PPV_base_ was significantly increased in cases of preload responsiveness than in cases of preload unresponsiveness. In twelve cases, the patients (*n* = 11) received fluids after performing all the tests, and in all of them, CI increased by more than 15%.

**Table 3:** Evolution of hemodynamic variables in preload responsive cases and non-responsive cases

Variables	EEO_6_		TVC		EEO_8_		TREND	
	Baseline	for PCCI_max_*	Baseline	for PPV_max_^†^	Baseline	for PCCI_max_*	Baseline	PCCI_max_*
*Heart rate (beats/min)*								
Responsive cases	89 ± 20	90 ± 20	90 ± 20	90 ± 21	90 ± 21	91 ± 21	90 ± 21	90 ± 19
Non-responsive cases	81 ± 20	81 ± 20	81 ± 20	81 ± 20	81 ± 20	80 ± 19	82 ± 20	80 ± 20
*Systolic arterial pressure (mmHg)*								
Responsive cases	122 ± 17	126 ± 18	126 ± 19	122 ± 18	121 ± 17	125 ± 18	122 ± 19	144 ± 20^abcd^
Non-responsive cases	125 ± 18	125 ± 18	124 ± 18	123 ± 17	124 ± 18	124 ± 19	124 ± 17	133 ± 18^abcd^
*Diastolic arterial pressure (mmHg)*								
Responsive cases	58 ± 8	59 ± 9	60 ± 9	58 ± 9	58 ± 8	59 ± 9	58 ± 9	70 ± 1^abcd^
Non-responsive cases	56 ± 9	56 ± 9	56 ± 9	55 ± 8	55 ± 9	55 ± 9	56 ± 9	62 ± 9^abcd^
*Mean arterial pressure (mmHg)*								
Responsive cases	79 ± 11	82 ± 12	82 ± 13	79 ± 11	78 ± 11	81 ± 11	79 ± 12	96 ± 14^abcd^
Non-responsive cases	80 ± 11	80 ± 11	79 ± 11	78 ± 11	79 ± 11	79 ± 11	79 ± 11	87 ± 12^abcd^
*Pulse pressure (mmHg)*								
Responsive cases	64 ± 14	66 ± 15	67 ± 15	64 ± 14	63 ± 14	66 ± 15	64 ± 15	74 ± 16^abcd^
Non-responsive cases	69 ± 16	70 ± 16	69 ± 16	68 ± 16	68 ± 16	69 ± 16	68 ± 15	71 ± 16^abcd^
*Central venous pressure (mmHg)*								
Responsive cases	8 ± 4	7 ± 4	8 ± 4	8 ± 4	8 ± 4	7 ± 4	7 ± 4	13 ± 5^abcd^
Non-responsive cases	11 ± 5	10 ± 5	11 ± 5	11 ± 5	11 ± 5	10 ± 5	10 ± 5	16 ± 8^abcd^
*Pulse pressure variation (%)*								
Responsive cases	9 ± 5^a^	8 ± 4^a^	9 ± 4^a^	13 ± 5^ab^	12 ± 5^ab^	11 ± 5^ab^	9 ± 4^ad^	9 ± 4^acd^
Non-responsive cases	5 ± 2	4 ± 2	4 ± 2	6 ± 3^b^	6 ± 3^b^	5 ± 3^b^	4 ± 2^d^	5 ± 3
*PCCI (L/min/m*^*2*^*)*								
Responsive cases	2.66 ± 0.75^a^	2.81 ± 0.78^a^	2.70 ± 0.73^a^	2.56 ± 0.66^a^	2.53 ± 0.65^a^	2.73 ± 0.69^a^	2.59 ± 0.67^a^	2.92 ± 0.74^abcd^
Non-responsive cases	3.35 ± 0.99	3.39 ± 1.00	3.33 ± 0.95	3.25 ± 0.94	3.27 ± 0.97	3.32 ± 0.97	3.43 ± 1.13	3.31 ± 1.01
*Intra-abdominal pressure (mmHg)‡*								
Responsive cases	11 ± 4	11 ± 4	11 ± 4	11 ± 4	11 ± 4	11 ± 4	11 ± 4	11 ± 4
Non-responsive cases	11 ± 4	11 ± 4	11 ± 4	12 ± 4	12 ± 4	11 ± 4^c^	11 ± 4	10 ± 4^ cd^

*EEO*_*6*_ end-expiratory occlusion performed at 6 mL/kg tidal volume, *EEO*_*8*_ end-expiratory occlusion performed at 8 mL/kg tidal volume, *Max* maximal value, *PCCI* pulse contour cardiac index, *TREND* Trendelenburg maneuver, *TV* tidal volume, *TVC* tidal volume challenge

*variables values collected for the maximal PCCI obtained during EEO_6_, EEO_8_ and TREND. ^†^variables values collected for the maximal PPV obtained during TVC. ^‡^available in 78 cases (40 responsive *vs.* 38 non-responsive cases). ^a^*p* < 0.05, responsive *vs.* non-responsive cases; ^b^*p* < 0.05 compared with EEO_6_ at the same timepoint; ^c^*p* < 0.05 compared with TVC at the same timepoint; ^d^*p* < 0.05 compared with EEO_8_ at the same timepoint

### *Performance of PPV*_*base*_*, ΔPPV TVC*_*6–8*_*, and ΔCI EEO*_*6*_* to assess preload responsiveness*

**Fig. 2:**
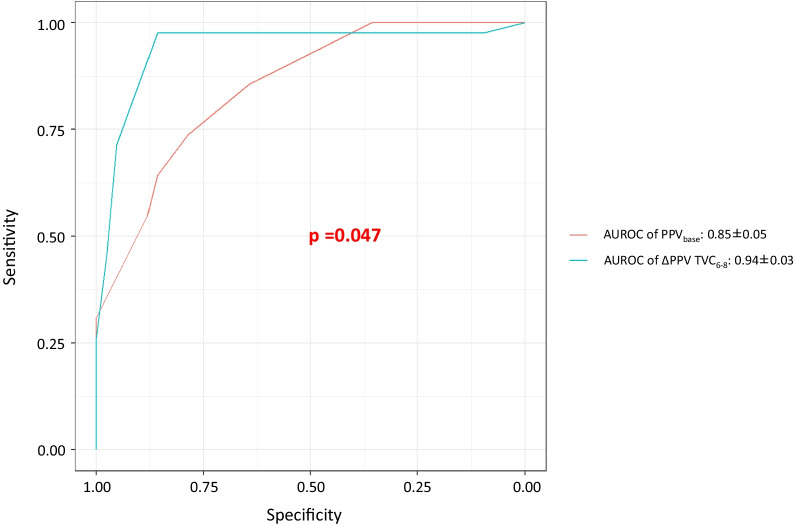
The comparison of the receiver operating characteristics (ROC) curves of baseline pulse pressure variation (PPV_base_) at a tidal volume of 6 mL/kg predicted body weight versus changes in pulse pressure variation during a tidal volume challenge (ΔPPV TVC_6–8_)

A PPV_base_ ≥ 6.5% enabled to assess preload responsiveness with an AUROC of 0.85 ± 0.05 (0.77–0.92), sensitivity: 74% (57–95%), specificity: 79% (56–96%), *p* < 0.01 versus 0.75; gray zone: 5–8% (32/84 cases) (Fig. 2, Additional file 1: Fig. S1). A detailed analysis of the 84 cases shows that all cases (*n* = 15) with PPV_base_ < 4% were non-responsive cases and that all cases (*n* = 13) with PPV_base_ ≥ 11% were responsive cases. In 56 cases, PPV_base_ was between ≥ 4 and < 11%.

A ΔPPV TVC_6–8_ ≥ 3.5% enabled to assess preload responsiveness with an AUROC of 0.94 ± 0.03 (0.88–0.99), sensitivity: 98% (89–99%), specificity: 86% (75–97%); *p* < 0.01 versus 0.75; *p* = 0.047 versus AUROC for PPV_base_; gray zone: 3.0–4.5% (28/84 cases) (Fig. 2, Additional file 1: Fig. S2). In the 56 cases where PPV_base_ was between ≥ 4 and < 11%, a ΔPPV TVC_6–8_ ≥ 3.5% enabled to assess preload responsiveness with an AUROC of 0.91 ± 0.05 (0.80–1.00), sensitivity: 97% (87–99%), specificity: 81% (65–96%), *p* < 0.01 versus*.* AUROC for PPV_base_: 0.66 ± 0.09 (0.49–0.83) (Additional file 1: Fig. S3).

An increase in CI ≥ 3.2% during EEO_6_ assessed preload responsiveness with an AUROC of 0.93 ± 0.06 (0.87–0.98), sensitivity: 88% (73–93%), specificity: 93% (90–99%); *p* = 0.911 versus AUROC for ΔPPV TVC_6–8_; gray zone: 2.2–4.6% (20/82 cases) (Additional file 1: Figs. S4 and S5).

### The effect of intra-abdominal pressure

In 34 cases, including 16 cases with preload responsiveness, IAP was ≥ 12 mmHg [43]. In these cases, PPV_base_ assessed preload responsiveness with an AUROC of 0.76 ± 0.09 (0.58–0.90); *p* = 0.90 versus 0.75 (Additional file 1: Fig. S6). A ΔPPV TVC_6–8_ ≥ 3% assessed preload responsiveness with an AUROC of 0.93 ± 0.05 (0.87–0.98), sensitivity: 97% (90–99%), specificity: 84% (63–99%); *p* < 0.01 versus 0.75; *p* = 0.06 versus AUROC for PPV_base_ (Additional file 1: Fig. S6). An increase in CI ≥ 3.2% during EEO_6_ assessed preload responsiveness with an AUROC of 0.93 ± 0.05 (0.83–0.99), sensitivity: 94% (68–99%), specificity: 87% (80–99%); *p* < 0.01 versus 0.75, *p* = 0.06 versus AUROC for PPV_base_; *p* = 0.90 versus AUROC for ΔPPV TVC_6–8_ (Additional file 1: Fig. S6).

## Discussion

Our study showed that in patients with ARDS under prone position, an increase in PPV ≥ 3.5% during one-minute TVC could reliably assess preload responsiveness and its predictive value was better than that of PPV alone.

The PPV is one of the most utilized dynamic indices to predict fluid responsiveness [44]. One of the reasons is that it can be easily obtained [21, 22]. However, interpretation of PPV is limited in many circumstances such as low tidal volume ventilation [25], arrhythmia, low respiratory compliance, and spontaneous breathing activity [24]. In patients who receive low tidal volume ventilation, respiratory changes in intrathoracic pressure might be insufficient to produce significant changes in preload and therefore PPV may lack sensitivity to predict fluid responsiveness [45]. This was illustrated by studies performed in patients with or without ARDS and who were mechanically ventilated with a tidal volume of ≤ 8 mL/kg [25, 46]. To compensate for the limitations of PPV at low tidal volume, it has been suggested to evaluate the response of PPV to a TVC [26]. An increase in PPV > 3.5% during a TVC was shown to predict fluid responsiveness reliably in patients who were ventilated with 6 mL/kg tidal volume in supine position (AUROC curve of 0.99) [26]. Another study [47] and a recent meta-analysis [20] have confirmed such excellent results in supine patients ventilated with low tidal volume. The findings of our present study confirmed that the TVC was still valid in patients with ARDS who underwent prone position with a threshold value (3.5%) which is the same as that previously reported [26]. The least significant change of PPV according to De Courson et al. was 8.9% [48]. Thus, for a given value of PPV, for example, 7 (which is the mean baseline value of PPV in our study), the smallest change in absolute value that can be trusted as a real PPV change would be small (less than 1 in absolute value). In our study, the cutoff value of absolute PPV change for TVC (+ 3.5) is thus higher than the least significant change value found in the previous literature [48].

Our study also confirmed that ΔPPV TVC_6–8_ could perform better than PPV_base_ to assess preload responsiveness. Importantly, there were many cases (56/84 cases) where PPV was inconclusive (between 4 and 11%) and where the ΔPPV TVC_6–8_ still reliably assessed preload responsiveness. The plateau pressure and hence the driving pressure increased significantly during the one-minute TVC, although there was no difference regarding respiratory compliance. Since the TVC is quite short, the effects on the driving pressure are expected to be transient and reversible. Nevertheless, caution should be taken in using this test in patients with markedly increased driving pressure.

Until now, only one study addressed the issue of assessment of preload responsiveness in patients with ARDS under prone position. Yonis et al. found that an increase in cardiac output greater than 8% during a Trendelenburg maneuver well assessed fluid responsiveness in a series of 33 patients under prone position and ventilated with 6 mL/kg tidal volume [28]. They also found that the changes in PPV during TVC did not well predict fluid responsiveness, which is in disagreement with our present results. It has to be noted that in this study [28], PPV and its changes in response to TVC were assessed in only 19/33 patients since 14 patients with cardiac arrhythmia were excluded from the analysis. There is no clear reason to substantiate the argument that heart–lung interactions cannot apply to patients in prone position as they apply to patients in the supine position. Therefore, there is no clear argument to support the fact that the hemodynamic effects of TVC are undermined during prone position. In this regard, in patients under prone position for neurosurgery and who received 6 mL/kg tidal volume, the response of PPV to a TVC was shown to predict fluid responsiveness with excellent accuracy (AUROC of 0.96; sensitivity: 95%; specificity: 95%) [27]. It has to be noted that the Crs was normal (around 65 mL/cmH_2_O on average) in the latter study, whereas it was low in the Yonis et al. study (around 30 mL/cmH_2_O on average), and this might account for the discrepancies between the findings of these two studies. Nevertheless, in the study by Myatra et al. [26], where the TVC performed very well to predict fluid responsiveness, the mean Crs was low (28 mL/cmH_2_O on average), and thus comparable with the Crs values reported by Yonis et al. [28]. In our present study, the Crs values were also low (around 30 mL/cmH_2_O on average) as we included patients with severe ARDS. An important difference between our study and that by Yonis et al. is the definition of preload responsiveness. As we did not administer fluids to all our patients with ARDS as they did, we defined preload responsiveness by the positivity of two preload responsiveness tests (Trendelenburg maneuver and EEO_8_). To minimize risks of uncertain interpretation, we excluded cases where one of the two tests was positive and the other one negative, a situation that occurred in 15% of cases. Therefore, in our study, we considered the presence of preload responsiveness when both ΔCI_TREND_ and ΔCI EEO_8_ were ≥ 8% and ≥ 5%, respectively, and the presence of preload unresponsiveness when both ΔCI_TREND_ and ΔCI EEO_8_ were < 8% and < 5%, respectively. It is noteworthy that our definition could identify the same proportion of preload responsive cases (50%) *vs.* preload unresponsive cases (50%) as reported in previous studies [16] including those where low tidal volume ventilation was used [20]. However, this does not totally exclude that both tests could be positive—according to our definition—while the patient would be fluid unresponsive and vice versa. Nevertheless, in all the cases with preload responsiveness—according to our definition—where fluid was administered, CI increased by ≥ 15% in response to fluid infusion, suggesting that our definition was appropriate at least in terms of specificity.

The predictive performance of PPV_base_ in our present study was better than that reported in some previous studies performed in patients receiving low tidal volume ventilation in supine position [25, 46]. Nevertheless, a recent meta-analysis that investigated the performance of PPV in patients under mechanical ventilation with tidal volume ≤ 8 mL/kg without arrhythmia and respiratory effort (22 studies) showed an AUROC of 0.82 (sensitivity 74% and specificity 77%) [20]. It is noteworthy that in patients with ARDS and ventilated with 6 mL/kg, Freitas et al. showed that PPV could predict fluid responsiveness with an AUROC of 0.91 (0.82–1.0), the sensitivity of 89%, and specificity of 90% [49]. Nevertheless, in our study, in two-thirds of cases, the PPV_base_ fell in a range of uncertainty (between 4 and 11%). Interestingly, the TVC might be helpful in the subgroup of cases where the intra-abdominal pressure was > 12 mmHg (*n* = 34) and where PPV_base_ failed to predict the preload responsiveness, although further studies are warranted to confirm this finding. All the above findings limit a broad application of PPV during prone position under low tidal volume ventilation and justify performing another test such as the TVC.

Since varied results were reported regarding the predictive performance of the EEO_6_ [26, 28, 50–52], we chose the EEO_8_ as one of the tests to definite the preload responsiveness in our current study in order to minimize uncertainty. Our results showed that the EEO test performed at 6 mL/kg tidal volume reliably assessed preload responsiveness in patients under prone position, which is consistent with our previous studies in the supine position [32, 51, 52] but in disagreement with some other studies showing a less reliable predictive performance of the EEO test at 6 mL/kg tidal volume in supine [19, 43] or in prone position [20, 21]. Nevertheless, although EEO_6_ seems to be reliable in prone position in our population, the cutoff value of CI change defining the preload responders was quite low (3.2%). Whereas the TVC only requires an arterial catheter to track the changes in PPV, the EEO_6_ has thus the disadvantage to require a real-time cardiac output monitor with a very high precision [31], a condition that is uncommon in resource-limited settings [53].

Our study has some limitations. Firstly, not all our patients received the standard fluid challenge, since administering fluid is not routinely performed by attending clinicians in patients with ARDS, even when preload responsiveness is present. Nevertheless, a postural maneuver (*i.e.*, PLR) had been previously used to replace fluid administration in order to evaluate the validity of preload responsiveness tests [54–56]. Secondly, to interpret the analysis more straightforwardly, we did not include the cases where only one of the two reference tests of preload responsiveness was positive (*i.e.*, either ΔCI_TREND_ ≥ 8% or ΔCI EEO_8_ ≥ 5%). This occurred, nevertheless in only 15% of cases. Thirdly, 16 patients were included more than once. It is noteworthy that these patients were never included twice during the same day or during the same prone position session. For these 16 patients, the delay between two inclusions was five days, which could be considered long enough for patients to present different hemodynamic profiles. Nevertheless, our statistical analysis took into account the effects of the repeated measurements on the same subject.

## Conclusion

In conclusion, the changes in PPV during a TVC can reliably assess preload responsiveness in patients with ARDS under prone position and low tidal volume ventilation. The advantage of this test, which was demonstrated to be superior to PPV, is that it does not require any cardiac output monitor to assess its effects.

## Supplementary Information

**Additional file 1: **Additional file on further results.

## Data Availability

The datasets used and/or analyzed during the current study are available from the corresponding author on reasonable request.

## Abbreviations

ARDS: Acute respiratory distress syndrome
AUROC: Area under the receiver operating characteristic
CI: Cardiac index
COVID-19: Coronavirus disease 2019
Crs: Compliance
CVP: Central venous pressure
EEO: End expiratory occlusion
FiO_2_: Fraction of inspired oxygen
IAP: Intra-abdominal pressure
ICU: Intensive care unit
PaO_2_: Arterial partial pressure of oxygen
PBW: Predicted body weight
PEEP: Positive end-expiratory pressure
PLR: Passive leg raising
PPV: Pulse pressure variation
ROC: Receiver operating characteristic
TPTD: Transpulmonary thermodilution
TVC: Tidal volume challenge
