# Supplementary material for: Tidal volume challenge to predict preload responsiveness in patients with acute respiratory distress syndrome under prone position
Source: Crit Care. 2022 Jul 18;26:219. doi: 10.1186/s13054-022-04087-w (PMC9294836; doi:10.1186/s13054-022-04087-w)
Supplement: Supplementary file 1 — Additional file 1: Additional file on further results. [file 13054_2022_4087_MOESM1_ESM.docx]

**Tidal volume challenge to predict preload responsiveness in patients with acute respiratory distress syndrome under prone position**

Rui Shi^1^, Soufia Ayed^1^, Francesca Moretto^1^, Danila Azzolina^2^, Nello De Vita^1^, Francesco Gavelli^1^, Simone Carelli^1^, Arthur Pavot^1^, Christopher Lai^1^, Xavier Monnet^1^, Jean-Louis Teboul^1^

^1^Université Paris-Saclay, AP-HP, Service de médecine intensive-réanimation, Hôpital de Bicêtre, Inserm S_999, Le Kremlin-Bicêtre, France

^2^Department of Environmental and Preventive Science, University of Ferrara, Ferrara, Italy.

**SUPPLEMENTAL FIGURES LEGEND**

Generalized Linear Mixed Model.

**Figure S1.** Gray zone (5%-8%) of baseline pulse pressure variation (PPV_base_) at a tidal volume of 6 mL/kg predicted body weight.

**Figure S2**. Gray zone (3.0%-4.5%) of changes in pulse pressure variation during a tidal volume challenge (ΔPPV TVC_6-8_).

**Figure S3.** The comparison of the receiver operating characteristics (ROC) curves of baseline pulse pressure variation (PPV_base_) at a tidal volume of 6 mL/kg predicted body weight *vs.* pulse pressure variation during a tidal volume challenge (ΔPPV TVC_6-8_) when the PPV_base_ are between 4% and 11%.

**Figure S4.** The comparison of the receiver operating characteristics (ROC) curves of baseline pulse pressure variation (PPV_base_) at a tidal volume of 6 mL/kg predicted body weight *vs.* pulse pressure variation during a tidal volume challenge (ΔPPV TVC_6-8_) *vs.* the end-expiratory occlusion (ΔCI EEO_6_) test at a tidal volume of 6 mL/kg.

**Figure S5.** Gray zone (2.2%-4.6%) of the end-expiratory occlusion (ΔCI EEO_6_) test at a tidal volume of 6 mL/kg.

**Figure S6.** The comparison of the receiver operating characteristics (ROC) curves of baseline pulse pressure variation (PPV_base_) at a tidal volume of 6 mL/kg predicted body weight *vs.* pulse pressure variation during a tidal volume challenge (ΔPPV TVC_6-8_) *vs.* the end-expiratory occlusion (ΔCI EEO_6_) test at a tidal volume of 6 mL/kg when the intra-abdominal pressure is higher than 12 mmHg.

**Generalized Linear Mixed Model**

A Generalized Linear Mixed model has been calculated to account for correlation within repeated measurements. The estimated models contain a random intercept term on the patient’s identification code with a fixed slope. The equation formula considered to estimate the model is as follows:

$$Y =\beta_{0j}+\beta_{1}group*\beta_{2}Condition$$

The variable $Y$indicates the study endpoint; the $\beta_{1}$ coefficient is the fixed effect term for the responsive versus non-responsive cases (GROUPS). Another fixed effect has been included in the model indicating the effect of the TESTS (EEO_6_, EEO_8_, TVC, Trendelenburg). An interaction term has been considered between GROUPS and TESTS.

The coefficient $\beta_{0j}$ is the random effect (intercept) term depending on the cluster $j$ patients ID.

The model has been estimated with lme4 [1] package in R [2].

**References**

1. Bates D, Mächler M, Bolker B, Walker S. Fitting Linear Mixed-Effects Models Using lme4. Journal of Statistical Software. 2015; 67:1 - 48.

2. R Development Core Team R: A Language and Environment for Statistical Computing.; R Foundation for Statistical Computing: Vienna, Austria, 2015.


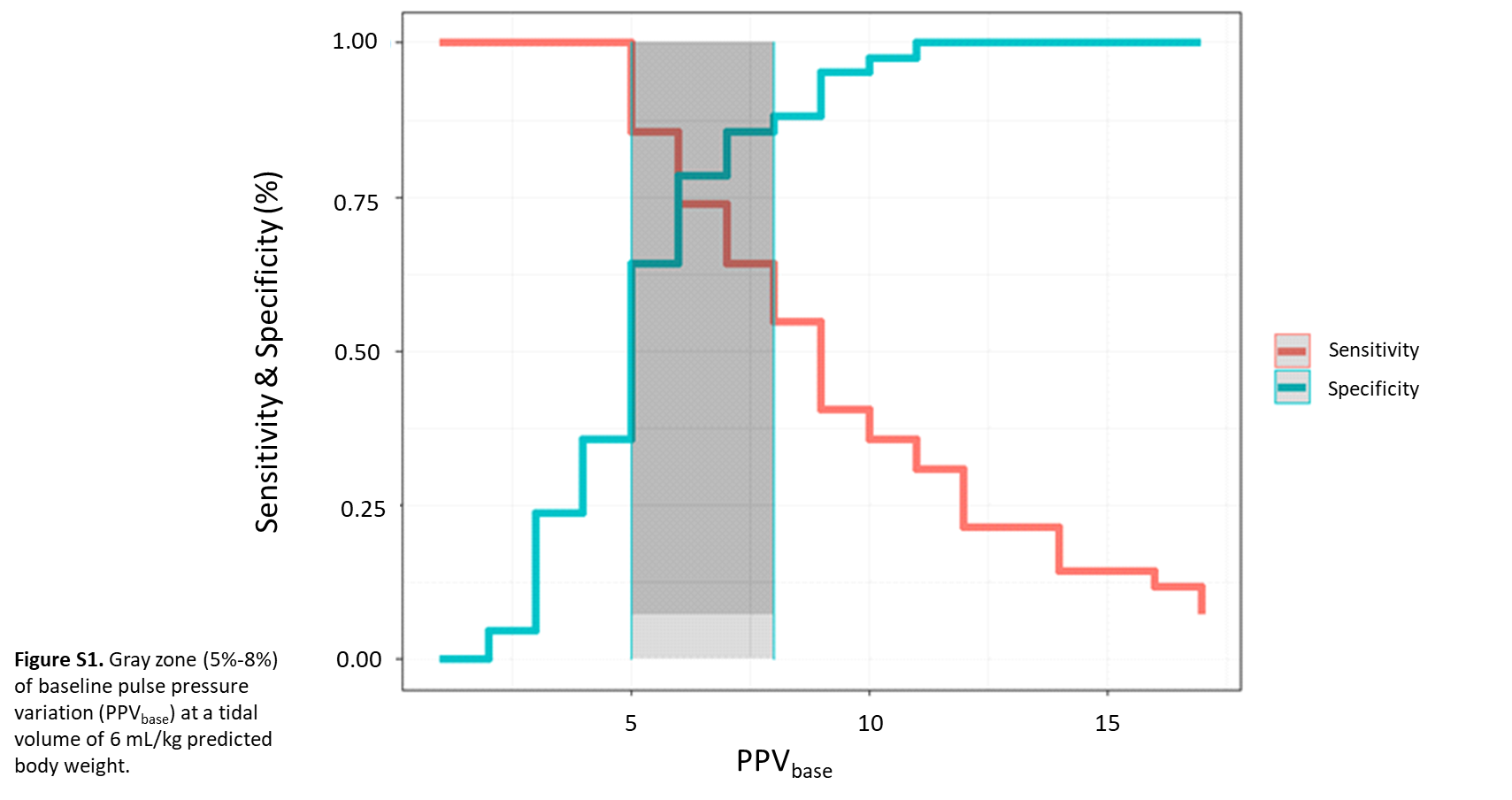


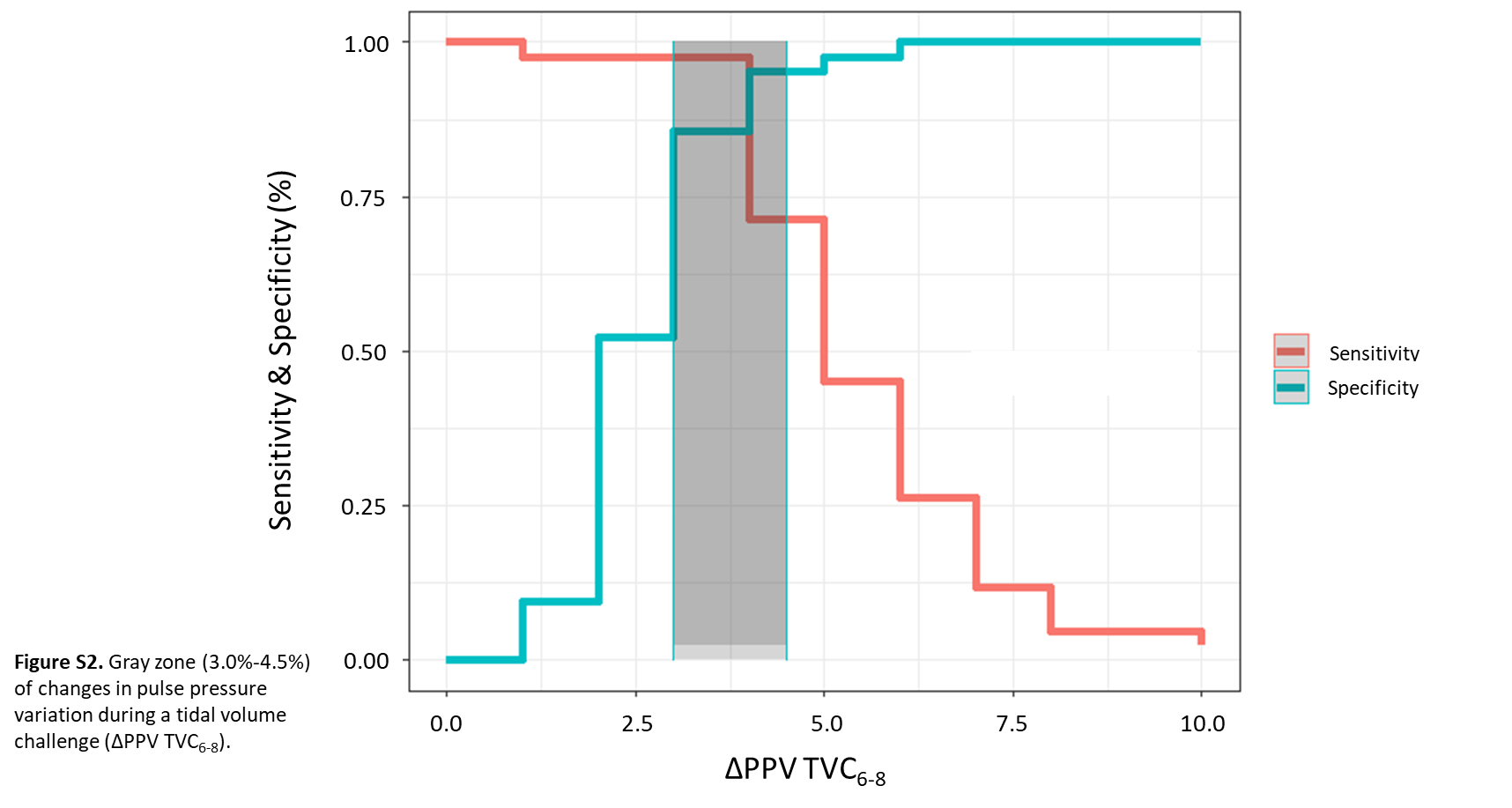


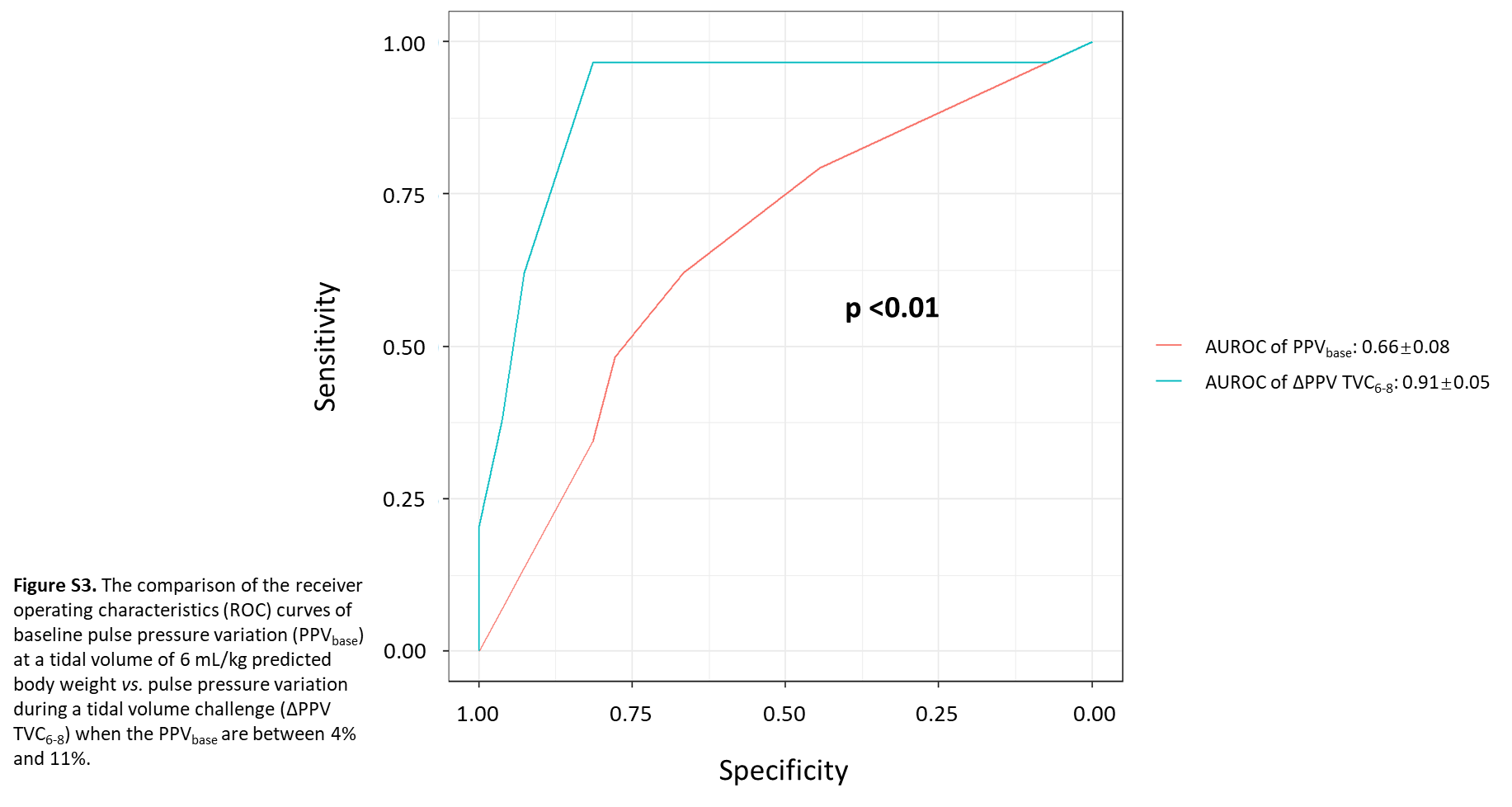


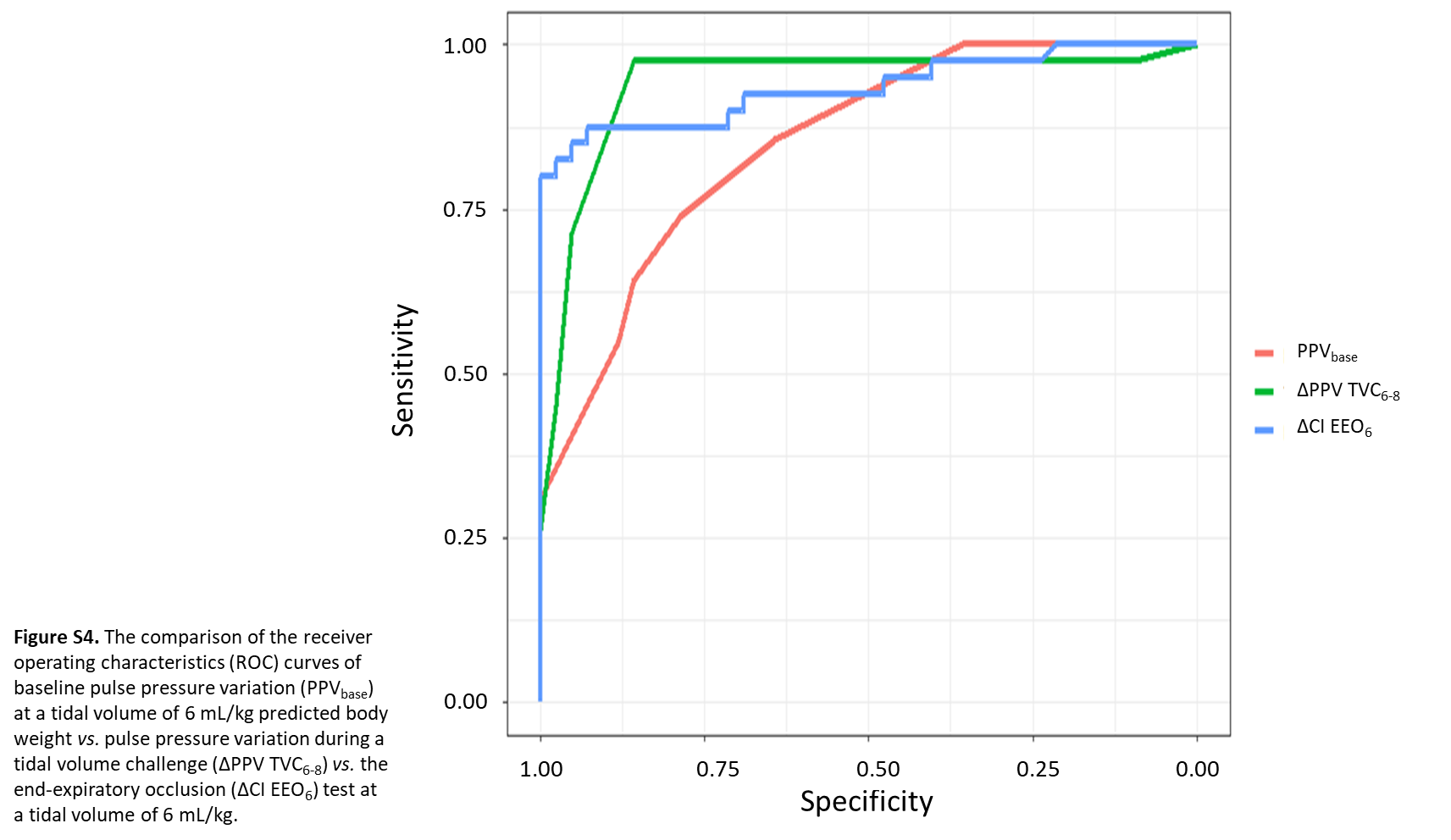


**
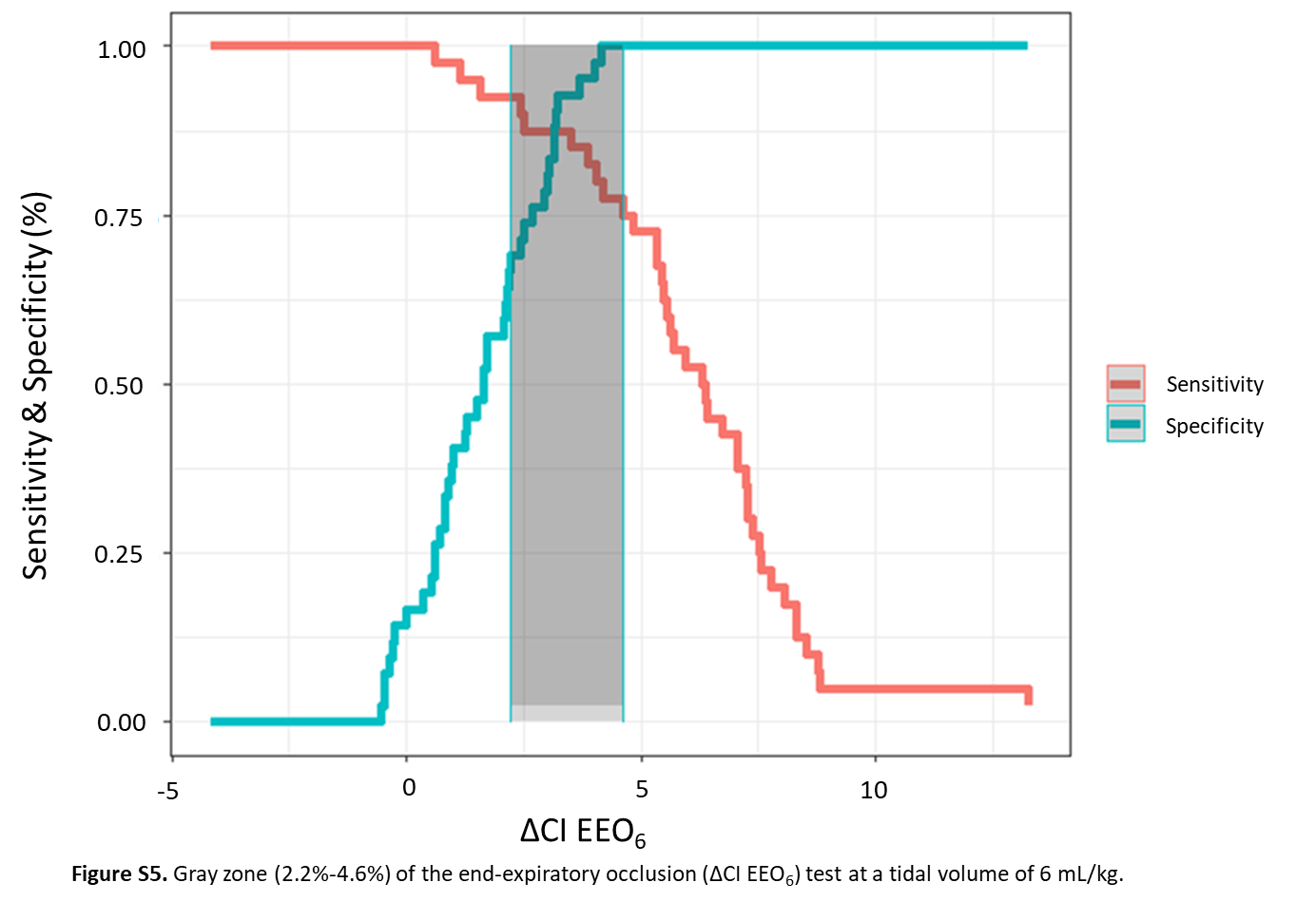
**

**
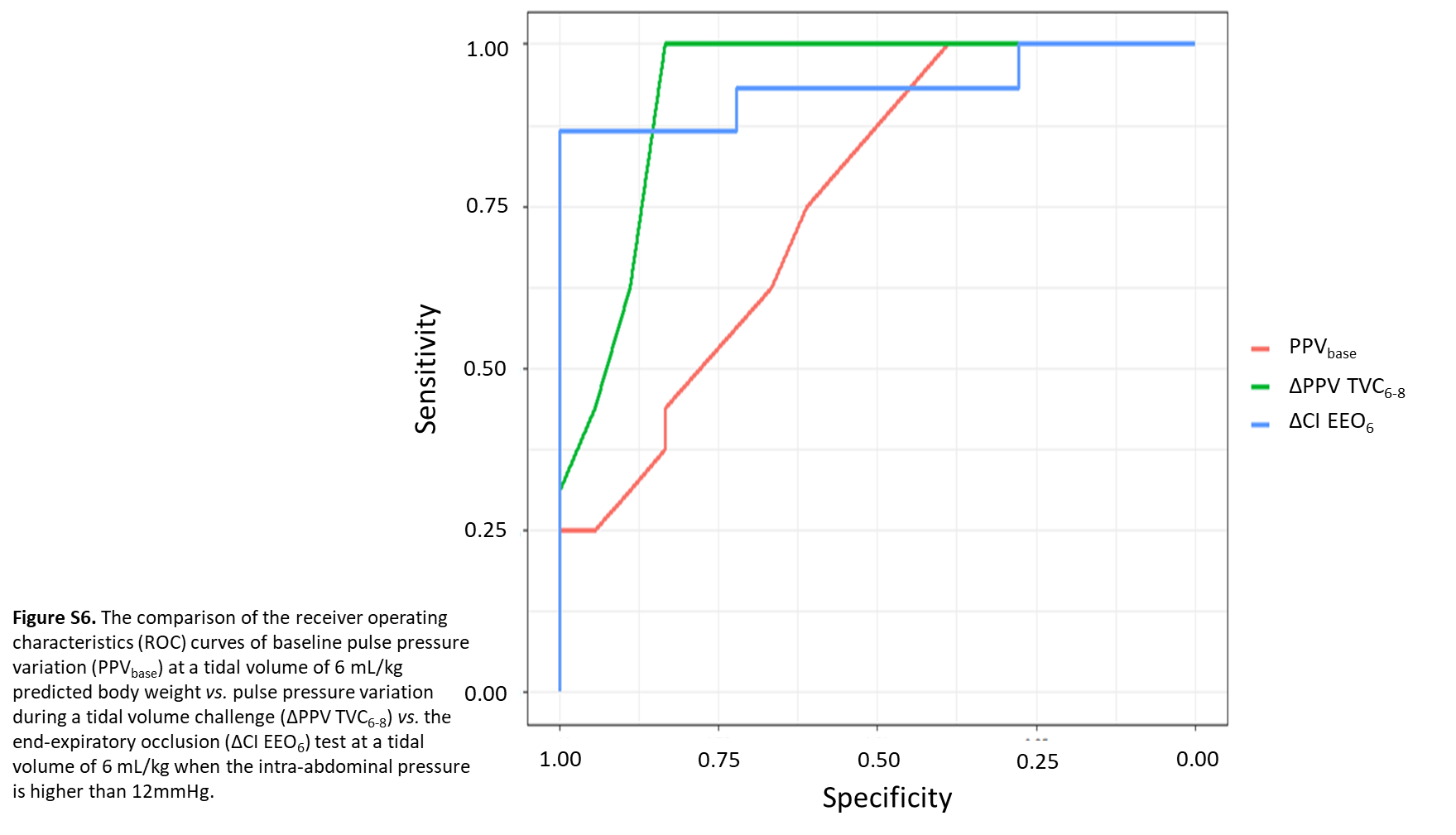
**
